# Two-Level Attention Module Based on Spurious-3D Residual Networks for Human Action Recognition

**DOI:** 10.3390/s23031707

**Published:** 2023-02-03

**Authors:** Bo Chen, Fangzhou Meng, Hongying Tang, Guanjun Tong

**Affiliations:** 1 Science and Technology on Microsystem Laboratory, Shanghai Institute of Microsystem and Information Technology, Chinese Academy of Sciences, Shanghai 201800, China; 2 School of Electronic, Electrical and Communication Engineering, University of Chinese Academy of Sciences, Beijing 100049, China

**Keywords:** action recognition, attention mechanism, spatiotemporal features, CNNs

## Abstract

In recent years, deep learning techniques have excelled in video action recognition. However, currently commonly used video action recognition models minimize the importance of different video frames and spatial regions within some specific frames when performing action recognition, which makes it difficult for the models to adequately extract spatiotemporal features from the video data. In this paper, an action recognition method based on improved residual convolutional neural networks (CNNs) for video frames and spatial attention modules is proposed to address this problem. The network can guide what and where to emphasize or suppress with essentially little computational cost using the video frame attention module and the spatial attention module. It also employs a two-level attention module to emphasize feature information along the temporal and spatial dimensions, respectively, highlighting the more important frames in the overall video sequence and the more important spatial regions in some specific frames. Specifically, we create the video frame and spatial attention map by successively adding the video frame attention module and the spatial attention module to aggregate the spatial and temporal dimensions of the intermediate feature maps of the CNNs to obtain different feature descriptors, thus directing the network to focus more on important video frames and more contributing spatial regions. The experimental results further show that the network performs well on the UCF-101 and HMDB-51 datasets.

## 1. Introduction

**Figure 1:**
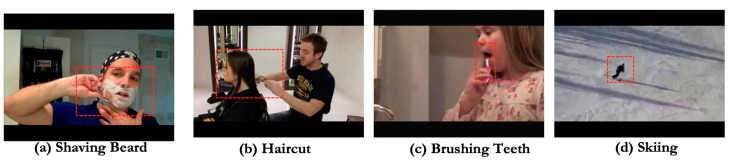
Examples of four video frames from the UCF-101 video dataset. From (**a**–**d**) all represent a video frame in which the action target occurs in a specific spatial region.

A great number of videos have been generated as a result of the advancement of multimedia technologies. In recent years, video understanding has emerged as a crucial field of computer vision for better analyzing and understanding video data. Human action recognition is one of the most fundamental tasks in video understanding, which is becoming increasingly popular in surveillance systems [1], health care systems, social robots [2], and other applications. In short, the ultimate goal of human action recognition is to allow machines to understand the action of the subject’s objectives in various observations, such as video frames through the camera sensors. Two important and complementary indications for action recognition in videos are spatial and temporal dynamics. The ability of a recognition system to extract and use useful information from it determines its performance to a considerable extent. However, owing to raw videos including far more redundant or irrelevant information in the space and time domains, extracting such information is tough. For example, in Figure 1, the discriminative part of four instances is just in the red dotted line box, demonstrating that not every part of the subject has a clear discriminatory effect on action recognition. As a result, it is critical to create effective representations to address these issues when learning categorical information about action classes.

**Figure 2:**
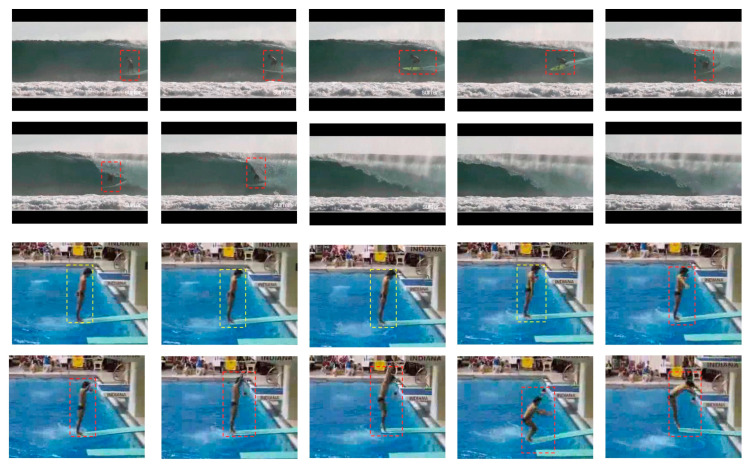
Two video examples from UCF-101 datasets.

The original video is cut into different video clips, each with different contributions to action recognition. Some clips have a discriminatory role in the classification, while others may lead to the action classifier being misled. For instance, as shown in Figure 2, the figures in the first and the second row are the same action, “Surfing”, but only the first seven frame figures contain the target of the action (marked by the red dotted line in the figure), while the surfer and the surfboard do not appear in the next few frame figures. These figures are irrelevant for recognizing the action. Although each figure in the third and the fourth row contains the action subject, the motion information of the several frames marked in the red box is not obvious. These figures appear in many actions of the same dataset, such as “Archery”, “Playing Flute”, “Swing”, “Jetski” and so on, which are not discriminative when it comes to recognizing action. When the action classifier averages the predictions from all figures, these irrelevant and non-discriminating figures deceive it. Previous approaches required pre-processing of input videos to remove irrelevant and non-discriminating figures. However, because pre-processing is normally performed by hand, it is not only time-consuming, but also requires significant financial resources in real-world applications.

Many studies on action recognition concentrate on modeling the temporal and spatial characteristics of the video. The key advantage of convolutional neural networks (CNNs) is their ability to extract spatiotemporal features directly from video frames in an effective manner. Inspired by the successful application of CNNs in image classification [3] and target detection tasks [4], CNNs have also been introduced into video-based human action recognition algorithm [5,6,7].

**Figure 3:**
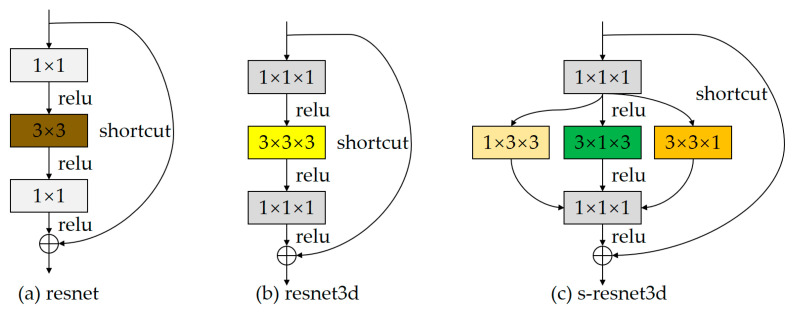
A “bottleneck” residual building block for resnet (**a**), resnet3d (**b**) and s-resnet3d (**c**). (**a**) shows the original residual network, (**b**) shows the 3d residual network after inflating the convolutional kernel, and (**c**) shows the spurious 3d residual network using 3 different convolutional kernels.

In our previous work [8], as shown in Figure 3a, which is the bottleneck of the original resnet [9], while in [10], the 3 × 3 convolution kernel is inflated to 3 × 3 × 3 for video action recognition, named resnet3d, and on this basis, we replace the 3 × 3 × 3 convolution kernels with 1 × 3 × 3, 3 × 1 × 3 and 3 × 3 × 1, respectively, and perform 2D convolution of the volumetric video data along the three views to learn collaborative spatiotemporal features by sharing the parameters of the different convolution kernels, named s-resnet3d. In order to address these challenges, we propose a video frame attention module that instructs the model to concentrate on the more relevant frames in the whole video by assigning different weights to different video frames, thus making it possible to avoid the negative effects of action categories of similarity between different video frames. Then, a spatial attention module is utilized for the more important frames mentioned above, so that the visual information of the action-related regions in the spatial features of the video frames is effectively captured, trying to eliminate the interference of negative information, such as noise and redundant information with action space features. For brevity, we name our model the frame and spatial attention network (FSAN). Finally, we implemented it in an end-to-end training way with ResNets and perform experiments on two datasets: UCF-101 and HMDB-51. The evaluation results show that our model is able to generate the state-of-the-art performance on the datasets using a similar backbone setup. The main contributions and novelty of this paper are summarized as follows:

- We propose an FSAN model with the ability to model spatiotemporal features of video information. FSAN contains a spurious-3D convolutional network and a two-level attention module that can be easily implemented and embedded into a CNN-based action recognition model with end-to-end training.
- We design an effective two-level attention module to help exploit information features across channel, time and space dimensions, and a video frame attention module to highlight the more important frames in the entire video sequence to reduce interference due to similarities between heterogeneous action video sequences. The spatial attention module focuses on the more important spatial regions in some given frames.
- Implementing end-to-end training on two challenging action recognition datasets, UCF101 and HMDB51, FSAN outperforms state-of-the-art video action recognition networks compared to existing methods.

The remainder of this paper is organized as follows. In Section 2, we first introduce classical video action recognition methods, from traditional manual features to deep learning-based approaches, and then describe related work on attention mechanisms used in the field of action recognition and beyond. In Section 3, we introduce in detail our proposed model and each of the modules. Section 4 describes the details of our experiments and evaluation and shows a comparison with state-of-art methods. It also includes an ablation study to verify module validity and determine the optimal module sequence. Section 5 concludes our work and points out future directions.

## 2. Related Works

Human action recognition in video has been widely used in areas such as autonomous driving, intelligent security, virtual reality, video parsing, military reconnaissance, sports training aids, etc. It has contributed significantly to the development and progress of many fields, both industrial and military, and has received considerable critical attention. In this section, we present the relevant action recognition models and attention mechanisms respectively.

### 2.1. Video-Based Action Recognition

Human action recognition is a hot topic in video understanding. Early work focused on designing a variety of handcrafted features to encode video data [11,12,13], particularly the Improved Dense Trajectories (IDT) [13], which dominated the field before deep learning was applied to the field because of its excellent results and robustness. However, handcrafted features are computationally expensive and difficult to scale and deploy, and the performance of these approaches is often limited.

With the rise of deep learning [14], the great success of convolutional neural networks (CNNs) in image recognition has driven researchers to start using CNNs for video problems, developing several deep learning-based methods to solve action recognition tasks. Simonyan et al. [15] proposed and used a two-stream model to train two separate convolutional networks: spatial, i.e., stream on single-frame RGB images to extract appearance features, and temporal, i.e., stream on multi-frame optical stream images to simulate motion features, and then fused their confidence scores to improve classification performance. Their experiments suggest that CNNs trained on dense frames of optical flow pictures can improve action recognition ability significantly. However, the optical flow computation will demand huge computational loads and memory resources. To mitigate these issues, Tang et al. [16] proposed a novel network named Temporal Segmentation Network (TSN), which uses a long-range modeling temporal structure to extract small clips from the videos based on a sparse temporal sampling strategy. The sampled video clips are then used as the input to the network. Each segment will obtain its initial prediction, understand the action category through the network, and then obtain a video-level prediction of the whole video through a consensus function of the segments. Another method of obtaining motion information is the 3D CNNs proposed by Tran et al. [10], which exploited the three-dimensional convolutional and pooling layers carried out on the large-scale video datasets in spatial and temporal domains simultaneously. Furthermore, several 3D CNNs variations have been developed; for example, Carreira et al. [17] built a unique two-stream inflated 3D CNNs (I3D) for learning spatiotemporal feature for video, which has the benefits and parameters of the 2D CNNs trained on ImageNet. I3D has achieved high performance in video recognition tasks because they can jointly capture the spatial and temporal information of the video, but each has its constraints. For instance, I3D cannot learn true spatiotemporal features since they employ late fusing of two streams classification scores, but 3D CNNs have high memory and processing demands. Given this, we intend to create an RGB-only CNN model for our action recognition work and achieve an architecture that can be trained in a video-level, end-to-end manner.

### 2.2. Attention Mechanism

The attention mechanism is used by the human visual system to aid with the efficient and effective analysis and interpretation of complicated situations. As a result, researchers have begun to incorporate attention processes into computer vision systems to increase their performance. In recent works, the visual attention mechanism has been widely applied to various CNNs-based models and yielded impressive results in both image [18,19,20,21] and video fields [22,23,24].

For the field of image recognition, Wang et al. [20] proposed attentional residual learning, which used residual connectivity to allow different layers of attentional modules to be fully learned, not only to focus operations on a specific region, but also to enhance the features of that part of the region. By combining feature channels as local models and fine-grained feature representations for joint learning, Zheng et al. [19] proposed a novel multi-attention convolutional neural network made up of convolutional, channel grouping, and part classification subnetworks. This allows part generation and feature learning to be mutually reinforced. In addition, recurrent neural networks (RNNs) and long short-term memory (LSTM) [25] units were utilized by Mnih et al. [26] and Vaswani et al. [21] to develop the attention-based model.

For the field of action recognition, by adaptively recalibrating channel feature responses, Chen et al. [27] proposed a Spatiotemporal Channel Attention Network that may effectively acquire distinguishing characteristics of human actions. Specifically, they built a Channel Attention Unit (CAU) module for STCAN, which is based on a two-stream architecture. The interdependencies across channels may be modeled using the CAU module to further provide weight distributions for boosting information properties in a targeted manner. In [28], Shi et al. investigated the visual attention process in video analysis and proposed a novel 3D-CNN model for learning attention-enhanced spatiotemporal representations. To acquire attention-enhancing features for improved spatiotemporal representation, they built an entity-enhanced regular learning module that leverages two-branch residual learning. In order to capture a wider variety of signals, Long et al. [29] developed a shifting operation in addition to a local feature integration framework based on attention clusters. There are also many works [30,31,32] on attentional mechanisms for video comprehension tasks.

In summary, with deep learning-based video human action recognition, as video data bring temporal information, we need to focus more not only on important features to reduce the interference of information such as noise in the spatial dimension but also on important frames in the temporal dimension. 

## 3. Module Design

**Figure 4:**
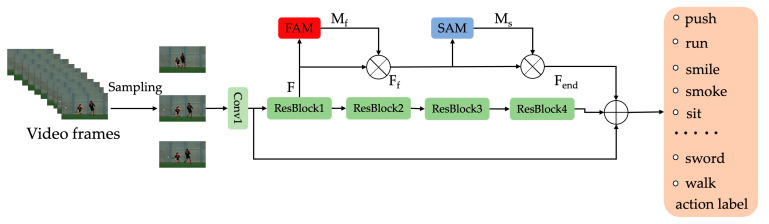
The overview of the FSAN.

In this section, we make a detailed description of the proposed FSAN. Figure 4 depicts the general structure of our model. In particular, it is noted that the ResBlock in Figure 4 is based on the s-resnet in Figure 3. We embed our proposed attention module on the last three residual blocks of the s-resnet. For convenience, we give a demonstration of only one ResBlock-connected attention module in Figure 4. In both the spatial and temporal dimensions, video often provides discriminating information for action recognition. However, the distribution of the identity data is frequently uneven. Not every component of the frame is directly connected to the activity in the spatial realm. For instance, in the action of hitting a box with a stick, the box and the stick should have more discriminatory information than the other pieces. Different frames do not convey an equal amount of information in the temporal domain. The video’s fewest frames include the most discriminating action recognition data. For instance, a golf swing is more discriminating than picking up the club. As a result, it is only normal to pay varying amounts of attention to various areas of the film. These discoveries led us to develop a frame and spatial attention module that can determine which portion of the video is more crucial.

### 3.1. Frame Attention Module

In video action recognition, each video frame contributes differently to the recognition of action categories. Some actions may occur in some concentrated frames, and some actions may occur in some long sustainable sequence of frames. Therefore, not all video frames are relevant to action recognition, and there are some video frames that are less relevant, or even not relevant at all, to the action category. If we feed these interfering frames into the network, this may introduce interference signals such as noise, making the recognition results inaccurate. Conversely, some frames are more relevant to the action category and more attention needs to be paid to these frames. The CNNs for human action recognition are more reliable when it concentrates on shorter but more information-rich sections of the action video rather than all of the video sequence. In order to learn the differentiation function, a frame attention module must be introduced. We compress the channel and spatial dimensions of the input feature map, retaining information in the temporal dimension to extract temporal features, and to obtain temporal descriptors, which are then used to efficiently aggregate frames consisting of information relevant to particular action categories and to generate frame attention scores. 

**Figure 5:**
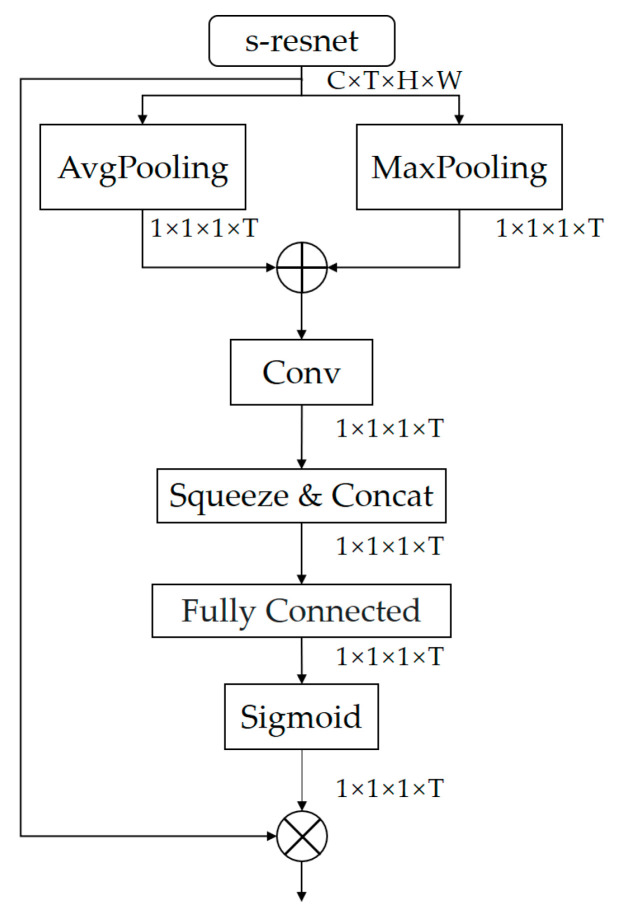
Frame attention module.

As shown in Figure 5. We obtain the output feature map of the s-resnet, mapping the original input feature map to $X_{t}^{\prime }=\left[x_{1}^{\prime },x_{2}^{\prime },x_{3}^{\prime },\;\ldots ,x_{t}^{\prime }\right]$. The initial features mapping $F\in R^{C\times T\times H\times W}$ is extracted to the s-resnet for spurious-3d convolution calculation and then fed to the video frame attention. This generated two distinct time feature descriptors, $Avg_{t}\in R^{1\times 1\times 1\times T}$ and $Max_{t}\in R^{1\times 1\times 1\times T}$, to represent the attention weights of the video frames. We concurrently compress the channel and spatial dimensions using AvgPool3d and MaxPool3d. The original features are then passed via the frame attention module to yield the frame attention map $Avg_{t}$ and $Max_{t}$. The complete operation is as follows:

(1) $${Avg}_{t}=AP3d\left(x_{t}^{\prime }\right)=\frac{1}{C\times H\times W}\overset{C}{\underset{c=1}{\sum }}\overset{H}{\underset{h=1}{\sum }}\overset{W}{\underset{w=1}{\sum }}{\mathbb{R}}^{C\times T\times H\times W}\;$$

and

(2) $$Max_{t}=MP3d\left(x_{t}^{\prime }\right)=\max\left\{X_{t}^{\prime }\right\}$$

where $Avg_{t}$ and $Max_{t}$ represent the set of global and local descriptors for the entire video, respectively; h and w represent the spatial indices, while c represents the channel indices; t is a temporal indicator in the range [1, 2, …, T]; C, H and W and denote the channel, height and width of the feature map, respectively. The module then aggregates two different temporal feature descriptors by element summation to obtain the final temporal feature descriptor $M_{f}\in R^{1\times 1\times 1\times T}$, which operates as follows:

(3) $$M_{f}=Avg_{t}+Max_{t}$$

Then, since the bottleneck of our s-resnet has three convolutions, we need to aggregate the feature maps of the three different convolutions, for which we used the function “cat”. Afterwards, we fed the integrated features into a fully connected (FC) layer.

Finally, the output was normalized using the sigmoid function. We can obtain the weights according to the following formula:

(4) $$w_{t}=\partial \left(Conv1D\left(M_{f}\right)\right)$$

where *Conv1D* denotes a one-dimensional convolution, ∂ is the sigmoid function and $\omega_{t}=R^{1\times 1\times 1\times T}$ in the range 0 to 1.

### 3.2. Spatial Attention Module

We may investigate spatial attention at the channel level, which aids in learning discriminative features for action recognition. The individual channels in a convolutional neural network-based model can be considered as spatially appearing representations of specific actions. We created a SAM for learning the relevance score of each channel of a convolutional neural network having a specific action feature, inspired by ECA-Net. The model emphasizes spatial regions with high scores related to particular action categories while suppressing regions with low values that are irrelevant. We feed the output feature map after the FAM module into the SAM module, which is efficiently compressed in both spatial and temporal dimensions to extract channel information and similarly obtain channel descriptors in order to efficiently capture spatial attention maps. Previous techniques have used global averaging pools such as ECA-Net and Se-Net to aggregate geographic information. It is noted in the literature [33] that using each pooling method individually is not as effective as using features from both the global average pool and the global maximum pool, the latter of which can significantly enhance the representational power of the convolutional neural networks.

**Figure 6:**
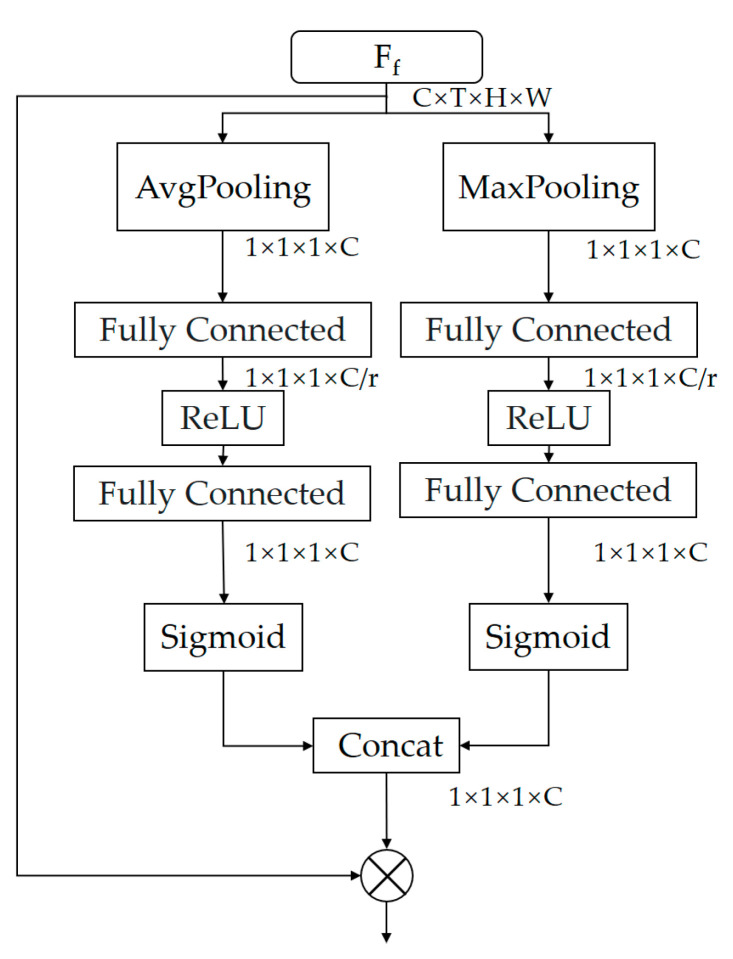
Spatial attention module.

Therefore, as shown in Figure 6, we use both the global average pool and the global maximum pool to aggregate the spatial and temporal dimensions of the video frame attention feature maps, and since channel information can be seen as a representation of spatial features, the channel dimension information is retained, and we end up with two different channel descriptions, i.e., $Avg_{s}$ and $Max_{s}$, respectively. The spatial attention feature descriptor can be obtained according to the following formula:

(5) $$M_{s}\left(F_{f}\right)=\partial \left\{w_{1}\left[w_{2}\left(Max_{s}\right)\right]+w_{1}\left[w_{2}\left(Max_{s}\right)\right]\right\}\;$$

where ∂ means the sigmoid function, which assures that the range of feature maps descriptor is from 0 to 1; w_1_ and w_2_ are trainable parameters, where r is the reduction ratio; $Avg_{s}$ and $Max_{s}$ are two descriptors, where $Avg_{s}$ counts the global background information for each channel and $Max_{s}$ counts the local discriminant information.

## 4. Experiments

All experiments in this paper are based on the Ubuntu 20.04 bionic operating system, with an Intel Xeon E5-2620v4 CPU and a GeForce RTX 2080 Ti GPU. On two benchmark datasets, we conduct extensive experiments in this section. The two benchmark datasets are briefly introduced initially, and then the specifics of the experiments—including data processing, training configuration and inference procedures—are presented. Some ablation studies are followed by several comparison experiments, which are then run and analyzed for our model and the state-of-the-art methods. All experiments were conducted on 4 GeForce RTX 2080 Ti GPUs using PyTorch (3.8).

### 4.1. Datasets

**Figure 7:**
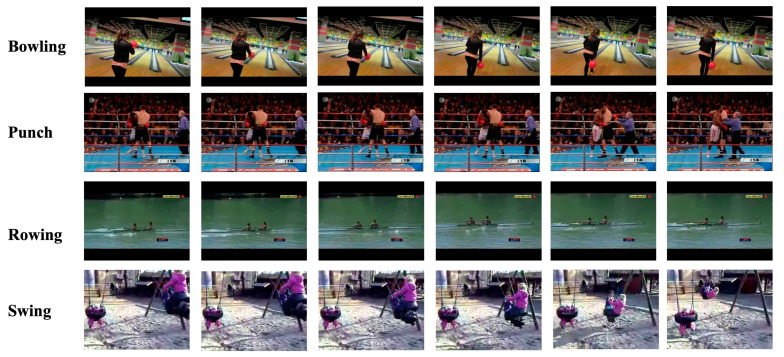
Frame examples of the UCF101 dataset.

**UCF101**: The UCF-101 dataset is an action recognition dataset made up of realistic-looking action videos. The dataset, which was compiled from YouTube, consists of 13,320 action video clips from 101 different human action categories. It also includes a range of difficult situations, including dim illumination, crowded backdrops and sharp fluctuations in camera movement. Non-action frames were briefly cut out of the videos. Each video clip is an average of seven seconds long and is broken down into the following five categories: (1) character interaction, (2) body movement, (3) human interaction, (4) playing an instrument and (5) human interaction. Typical action examples in the UCF-101 datasets are shown in Figure 7. This dataset is randomly spilt into three subdatasets, 70% of which are used to train and 30% for testing.

**Figure 8:**
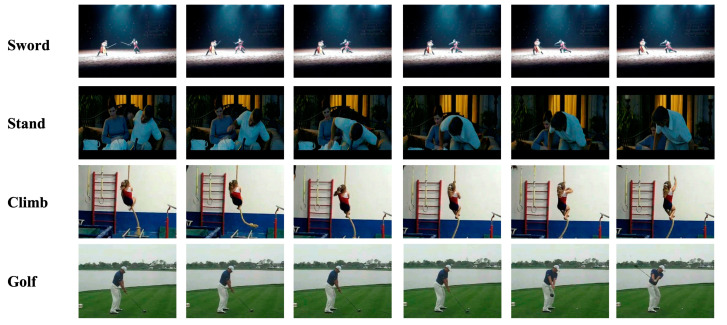
Frame examples of the HMDB51 dataset.

**HMDB51**: The HMDB-51 dataset contains 6766 clips divided into 51 action categories. There are at least 101 clips in each action class, largely from movies but also a few from online video repositories, such as YouTube and Google Video. The dataset faces the difficulty of greater intra-class and lesser inter-class heterogeneity. The average length of the video snippets was 3 s, just like in UCF-101. Similar to UCF-101, the training/test split is used. Actions fall into one of five categories: (1) general facial actions, (2) facial actions involving object manipulation, (3) general body actions, (4) body interactions involving objects and (5) body interactions involving other people. Typical action examples in the HMDB-51 are shown in Figure 8. This dataset provides three subdatasets, 70% of which are used to train and 30% for testing.

### 4.2. Implementation Details

#### 4.2.1. Data Processing

Using quick video-loading libraries (such as decord and PyAV), we decoded the video more quickly to produce the original frames. Using the decord tool, we extracted the original frames from the movie, sampled them to get 64 consecutive frames and then extracted 8 frames at 8-frame sample intervals. We fed the network clips that are 3 channels by 16 frames by 224 pixels by 224 pixels in size. We employed techniques for data augmentation that are comparable to those in [34]. Random cropping, random flipping at a flip ratio of 0.5, and other data improvement techniques were used.

#### 4.2.2. Training Settings

On a four-GPU system, we trained our model 100 epochs. With a momentum of 0.9 and a weight decay of 0.0001, we used stochastic gradient descent (SGD) to train our network model. Our network model was trained from scratch using two datasets. The learning rate was 0.002 at first. We chose dropout with a dropout ratio of 0.5 after the global pooling layer and discovered that activating batch normalization in our application decreases overfitting. In our application, we discovered that turning on batch normalization lessens overfitting. To initialize the weight layers, we employed the technique from [35].

#### 4.2.3. Inference

We evaluated the proposed model on two public benchmarks (UCF101 and HMDB51). Then, we extracted 10 clips from the original videos and calculated their action recognition scores in the time domain. The largest classification score indicates the corresponding class label. Finally, we calculated the average of all segment classification scores and recorded it as the result of the test.

### 4.3. Ablation Studies

First, we performed comparative experiments with the baseline without any attention module and with the addition of a frame attention module and a spatial attention module, respectively, with the particular note that the baseline used in the experiments in this paper was s-resnet and the dataset used for the experiments was HMDB-51 (Split1). These two modules are capable of being connected in many ways (in parallel or in cascade). In addition, the sequence between the two modules can be swapped at will. Through ablation studies, we have concluded that the optimal architecture of FSAN is that the spatial attention module is followed sequentially by the video frame attention module.

**The Validation of FAM and SAM.** We denote the baseline network as s-resnet50, the network with only the spatial attention module embedded is noted as SAM and, similarly, the network with only the video frame attention module embedded is noted as FAM. We compared the performance of the three in HMDB-51. We investigated three ways of combining models: parallel and fused (F//S), sequential spatial-frame (SF) and sequential frame-spatial (FS).

As a result of Table 1, whether the two modules are embedded in the network alone or both, they are useful for action recognition, and they improve performance at both baselines. SAM, and FAM were significantly higher than the baseline by 1.2% and 1.7%, respectively. These results indicate that attention to spatial regions is highly correlated with action categories, and keyframes comprising action categories can enhance the prediction robustness of the model. The frame attention module has higher accuracy than the spatial attention module. This suggests that temporal attention plays a dominant role by selecting keyframes that are relevant to the action category rather than reducing the interference of irrelevant frames on recognition performance.

**The Arrangement of FAM and SAM.** We investigate three ways of combining models: parallel and fused (F//S), sequential spatial-frame (SF) and sequential frame-spatial (FS). Here, SF means that the spatial attention module precedes the video frame attention module, and conversely, FS means that the video frame attention module precedes the spatial attention module. It can be verified whether assigning spatial weights first is more helpful for action recognition or assigning video frame weights first is more helpful for action recognition.

As seen in Table 2, sequential Frame-Spatial (FS) had the best performance, improving by 5.2% and 4.7% over SAM and FAM, respectively. Parallel optimization is more difficult than sequential optimization. The above results validate the complementary nature of SAM and FAM. The model combines the advantages of SAM and FAM to further improve the recognition performance of the CNN-based action recognition method. Moreover, our model is able to focus on keyframes containing action categories while highlighting the spatial regions associated with the acting categories, facilitating the robustness of the model in extracting features. Our module contains an attention module for temporal sequencing and is consistently applied in all experiments.

### 4.4. Comparisons with the State-of-the-Art

We compare the performance of our FSAN architecture with other state-of-the-art methods on two action recognition benchmark datasets: UCF-101 and HMDB-51. On the UCF101 and HMDB51 datasets, we fine-tuned the network pre-trained on Kinetics400 or Imagenet. To be fair, we considered an approach with only RGB frames without any additional modalities (e.g., optical flow and multiscale testing) as input. The results are shown in Table 3. In the pre-trained column of Table 3, ‘-’ indicates that no pre-training was performed. Our method outperforms other state-of-the-art methods with the same backbone on all datasets. Although MEST and R(2 + 1)D outperform our FSAN, they have approximately two to three times more network parameters than FSAN, because they use more network streams to extract relevant features.

As shown in Table 3, on the UCF-101 dataset, our FSAN model showed a significant improvement of about 4% compared to other models, except for the MEST model and R(2 + 1)D, which fully demonstrates the effectiveness of our proposed model. Meanwhile, by pre-training on the Kinetics400 dataset, our model improved over the original one.

As listed in Table 3, on the HMDB-51 dataset, our FSAN model has a significant advantage over IDT, TSN and DANet. Again, our model improves on the original by approximately 10%. This greatly illustrates the great superiority of our model in action recognition. Our network performs better on UCF101 and weaker on HMDB51 compared to DB-LSTM, perhaps because the LSTM is better at recognizing movie clips. The HMDB51 dataset consists mainly of movie clips, while the UCF101 dataset consists mainly of sports clips.

Our best results outperformed many methods on the HMDB-51 dataset and the UCF-101 dataset, demonstrating the importance of attention mechanisms and the effectiveness of the spatial attention module and frame attention module. In terms of action recognition, our model can distinguish spatiotemporal feature representations, highlight action categories with in-frame and focus on keyframes associated with action categories through space and time. Furthermore, on both datasets, our model also outperforms the latest attention-based approaches such as STA [40]. However, the R(2 + 1)D [42] and MEST [44] networks achieve better performance than our method on the UCF-101 dataset. This is because these methods use expensive optical flow maps in addition to RGB input frames. The optical flow needs to be extracted from the image in advance, which is usually computationally intensive and therefore difficult to obtain for large-scale datasets. Although our accuracy is lower compared to TDN [47], our model parameters are much lower, which is an acceptable result.

## 5. Conclusions

In this paper, a new stacked diverse attention network is proposed. The method uses a 3D CNN to extract basic deep features and then mines discriminative features between actions using the proposed attention model. To better focus on key information and keyframes in the feature graph, a spatial attention module is designed, which is an attention mechanism that gives higher importance scores to spatial regions and video frames that are more relevant to the action category without dimensionality reduction through a local cross-channel interaction strategy. State-of-the-art performance can be achieved in action recognition tasks, and extensive experiments demonstrate the effectiveness of the proposed FSAN. In future research, we would like to investigate how to improve the robustness of the attention mechanism to achieve better performance.

## Figures and Tables

**Table 1:** The validation of the attention module, embedding module into baseline.

	Model	HMDB-51
Baseline	s-resnet50	66.2%
Spatial	SAM	67.4%
Frame	FAM	67.9%

**Table 2:** Comparing the performance of our model with different sequences.

	Model	HMDB-51
Both	F//S	68.4%
	SF	69.2%
	FS	72.6%

**Table 3:** Comparison of the state-of-the-art on UCF-101 and HMDB-51 datasets with only RGB frames as inputs.

Method	Pre-Trained	Params(M)	UCF-101	HMDB-51
IDT [13]	-	-	86.4%	61.7%
Two-stream [15]	ImageNet	25	88.%	59.4%
C3D [10]	Kinetics400	78	85.2%	-
TSN [16]	ImageNet	24.3	94%	68.5%
P3D [36]	Kinetics400 + ImageNet	25.4	88.6%	-
MiCT-Net [37]	Kinetics400	50.2	88.9%	63.8%
STA [38]	-	35.3	89.5%	70.2%
STA-TSN [39]	-	29.8	82.1%	51%
DANet [40]	-	36.26	86.7%	54.3%
R(2+1)D [41]	Kinetics400	63.6	96.8%	74.5%
DPF [42]	-	48.6	79.6%	-
MEST [43]	ImageNet	89.32	96.8%	73.4%
FSTFN [44]	-	39	92.4%	69.43%
ActionS-ST-VLAD [45]	-	-	95.6%	71.4%
TDN [46]	Kinetics400 + ImageNet	52.3	97.4%	76.3%
Multi-Domain [47]	Kinetics400	32.02	94.82%	71.57%
TCLR [48]	ImageNet	45	82.4%	52.9%
DB-LSTM [49]	-	-	91.21%	87.64%
HAR-Depth [50]	-	-	92.97%	69.74%
Ours	Kinetics400	30.12	95.68%	72.6%

## Data Availability

The data that support the findings of this study are available from the corresponding author upon reasonable request.
